# Identification of functionally important microRNAs from rice inflorescence at heading stage of a *qDTY4*.*1*-QTL bearing Near Isogenic Line under drought conditions

**DOI:** 10.1371/journal.pone.0186382

**Published:** 2017-10-18

**Authors:** Boon Huat Cheah, Sudhir Jadhao, Madavan Vasudevan, Ratnam Wickneswari, Kalaivani Nadarajah

**Affiliations:** 1 School of Biotechnology and Bioscience, Faculty of Science and Technology, Universiti Kebangsaan Malaysia, Bangi, Selangor, Malaysia; 2 Bionivid Technology Private Limited, NGEF East Kasturi Nagar, Bangalore, India; 3 School of Environmental and Natural Resource Sciences, Faculty of Science and Technology, Universiti Kebangsaan Malaysia, Bangi, Selangor, Malaysia; Dokuz Eylul Universitesi, TURKEY

## Abstract

A cross between IR64 (high-yielding but drought-susceptible) and Aday Sel (drought-tolerant) rice cultivars yielded a stable line with enhanced grain yield under drought screening field trials at International Rice Research Institute. The major effect *qDTY4*.*1* drought tolerance and yield QTL was detected in the IR77298-14-1-2-10 Backcrossed Inbred Line (BIL) and its IR87705-7-15-B Near Isogenic Line (NIL) with 93.9% genetic similarity to IR64. Although rice yield is extremely susceptible to water stress at reproductive stage, currently, there is only one report on the detection of drought-responsive microRNAs in inflorescence tissue of a *Japonica* rice line. In this study, more drought-responsive microRNAs were identified in the inflorescence tissues of IR64, IR77298-14-1-2-10 and IR87705-7-15-B via next-generation sequencing. Among the 32 families of inflorescence-specific non-conserved microRNAs that were identified, 22 families were up-regulated in IR87705-7-15-B. Overall 9 conserved and 34 non-conserved microRNA families were found as drought-responsive in rice inflorescence with 5 conserved and 30 non-conserved families induced in the IR87705-7-15-B. The observation of more drought-responsive non-conserved microRNAs may imply their prominence over conserved microRNAs in drought response mechanisms of rice inflorescence. Gene Ontology annotation analysis on the target genes of drought-responsive microRNAs identified in IR87705-7-15-B revealed over-representation of biological processes including development, signalling and response to stimulus. Particularly, four inflorescence-specific microRNAs viz. osa-miR5485, osa-miR5487, osa-miR5492 and osa-miR5517, and two non-inflorescence specific microRNAs viz. osa-miR169d and osa-miR169f.2 target genes that are involved in flower or embryonic development. Among them, osa-miR169d, osa-miR5492 and osa-miR5517 are related to flowering time control. It is also worth mentioning that osa-miR2118 and osa-miR2275, which are implicated in the biosynthesis of rice inflorescence-specific small interfering RNAs, were induced in IR87705-7-15-B but repressed in IR77298-14-1-2-10. Further, gene search within *qDTY4*.*1* QTL region had identified multiple copies of NBS-LRR resistance genes (potential target of osa-miR2118), subtilisins and genes implicated in stomatal movement, ABA metabolism and cuticular wax biosynthesis.

## Introduction

Rice (*Oryza sativa*) together with wheat and maize provides half of the total calories consumed by the entire world population. Of that total calorie, 23% is provided by rice, 17% by wheat and 9% by maize [1]. Rice is widely grown in Asia, where 90% of the world’s rice grown here is for local market consumption [2,3]. The 158 million hectares of annual harvested rice area is made up of various agricultural systems, including irrigated (50%), rainfed lowland (34%), rainfed upland (9%) and deepwater (7%) [4]. Rice agriculture requires 2–3 times more water than wheat or maize owing to well-puddled and irrigated conditions required for cultivation [4,5]. It is estimated that irrigated rice consumes between a quarter to a third of the world’s tapped freshwater resources [6]. The world population increases by 75 million people a year and is projected to reach 8.8 billion by the year 2050 [7]. The increasing competition for global land and water resources between rice agriculture and other socioeconomic activities, such as urbanization, industrialization or biodiversity protection, being imminent [8,9], has increased the pressure to reduce water use in the water-intensive irrigated rice production. Rice is highly vulnerable to water stress and attempts to lower water input to rice fields may compromise its yield [10]. Further, water scarcity in rice planting is aggravated by the on-going process of climate change which causes irregular rainfall patterns in cropping seasons, resulting in widespread drought in rice-growing areas leading to severe yield losses [11]. Water shortage is, therefore, one of the most critical challenges facing rice agriculture nowadays, with a total of 23 million hectares of rice fields affected by drought at variable intensities [12]. In order to meet the growing population’s food demands, higher rice yield must be achieved from less land and water. Hence, breeding rice varieties with increased yield under drought could be the answer to this challenge. Realising its importance, the International Rice Research Institute (IRRI) continues efforts in breeding drought-tolerant varieties namely Sahod Ulan and Sookha dhan, which have been released and are currently being cultivated by farmers in Philippines and Nepal respectively [12].

Over the past decade, drought breeding research at IRRI has focused on direct phenotypic selection on grain yield in dry environments as it is an effective criterion for improving drought tolerance in rice [13]. A cross of the high-yielding but drought-susceptible rice cultivar IR64 and the traditional, drought-tolerant cultivar Aday Sel resulted in the identification of a BIL, IR77298-14-1-2-10 [14]. Under drought screening field trials at IRRI, IR77298-14-1-2-10 consistently showed a higher grain yield under drought stress (1183kg ha^-1^) compared to IR64 (342kg ha^-1^) [5]. A major-effect QTL for drought tolerance and increased yield, *qDTY4*.*1*, was detected in IR77298-14-1-2-10/IR64 population which explained 11.2% phenotypic variation for grain yield [15]. The *qDTY4*.*1* is located between markers RM335-RM518 with QTL peak marker being RM518 on chromosome 4 [15]. As this QTL was detected by genetic mapping for yield under drought, information on its physiological and molecular drought responses was not available [14]. For the characterization of physiological and molecular drought responses controlled by *qDTY4*.*1*, near-isogenic line (NIL) bearing *qDTY4*.*1*, namely IR87705-7-15-B, with 93.9% genetic similarity to IR64, was generated [14]. In this study, three rice lines (IR64, IR87705-7-15-B, IR77298-14-1-2-10) were used to better understand the molecular mechanism of drought response through expression profiling of microRNAs (miRNAs). In addition to IR77298-14-1-2-10 and IR87705-7-15-B that are known to have improved yield performance under drought, two genotypes (i.e. IR64 and IR87705-7-15-B) with high genetic similarities and contrasting yields under drought were compared. Heterogeneous genetic backgrounds of tolerant and susceptible germplasms often complicate the finding of genes that truly determine drought tolerant phenotype due to false positive discoveries [16]. Therefore, the choice of lines in this study may lead to new discoveries of the genetic modulation in drought response circuitry.

The expression profiling in this study focuses on miRNAs, a large class of 21–24 nucleotide (nt) small non-coding RNAs that regulate gene expression at post-transcriptional level in eukaryotes [17,18]. In plants, miRNAs mainly mediate gene silencing of their highly complementary target messenger RNAs (mRNAs) by guiding Argonaute (AGO) proteins to cleave the target mRNAs [19,20]. miRNAs can be classified into different families where each miRNA family may consist of highly identical mature miRNA members [20,21]. These miRNA families can be divided into ancient and young miRNAs. The former are generally abundantly expressed and conserved in different plant species, while the latter are often weakly expressed or only accumulate under certain environmental conditions and present only in limited species, making them the non-conserved miRNAs [22,23]. While conserved families are well known to predominantly regulate transcription factors, the biological functions of non-conserved miRNAs have not been well documented primarily due to the lack of functional targets. Despite this, the observation of non-conserved miRNAs that are expressed spatially or induced under certain conditions may point us to their plausible regulatory roles in plant physiology [23]. In rice, there are 21 conserved miRNA families and approximately 400 non-conserved miRNA families of which most of them have only single miRNA members.

Various developmental processes, namely maintenance of shoot apical meristems [24,25], initiation of axillary shoot meristems [26], leaf [27,28], root [29,30] and floral [31] developmental control are controlled by miRNAs [32]. miR156 and miR172 are two well-studied miRNAs in floral control. miR172 was reported to control flowering time and floral organ identity in both monocotyledons and dicotyledons with differing morphological outcomes in different species [31]. Its over-expression in *Arabidopsis* down-regulates the plant-specific *AP2* (floral organ identity gene) and *AP2*-like (floral repressors) transcription factor genes through translational repression that respectively causes *ap2* mutant flower phenotype and early flowering [33,34]. miR156 acts upstream of miR172 by repressing SQUAMOSA promoter binding protein-like (*SPLs*) transcription factor genes, whose encoded products are the transcriptional activators of miR172. The involvement of these miRNAs in controlling floral development indicates that there is possibly a group of miRNAs that modulates drought responses in rice inflorescence. However, previous drought studies of rice miRNAs have either been conducted on seedlings [35–37] or restricted to leaf tissues of different developmental stages (tillering and inflorescence formation) [38]. Nevertheless, detection of drought-responsive miRNAs at reproductive stage is crucial as floral fertility in rice is very sensitive to water stress at the expense of yield [13,39]. To date, Barrera-Figueroa’s research group has detected 18 drought-responsive miRNAs in *Japonica* rice inflorescences [40] indicating the need to further study miRNA expression in reproductive tissues under drought stress.

In this study, Illumina sequencing technology was chosen to profile drought-responsive miRNAs in inflorescence tissues of rice lines with contrasting levels of drought tolerance. This high-throughput sequencing allows the detection of lowly expressed miRNAs (especially non-conserved miRNAs) which are difficult to detect by other expression profiling methods. Overall, our results highlighted 22 families of inflorescence-specific non-conserved miRNAs found as induced in the inflorescence of the IR87705-7-15-B under drought treatment. Functional annotation of their predicted targets showed over-representation of three biological processes, namely development, response to stimulus and signalling. In particular, the putative target genes of 4 inflorescence-specific miRNA families were annotated as candidates for controlling flower or embryonic development.

## Materials and methods

### Plant materials

Three rice lines with contrasting levels of drought tolerance: IR77298-14-1-2-10 (drought-tolerant; BIL) [5], IR87705-7-15-B (drought-tolerant; NIL) [14] and IR64 (drought susceptible) [5] were selected. They were planted in pots placed in greenhouse under natural solar irradiance. Sufficient watering to rice plants was given up to booting stage. Each rice line was divided into two experimental conditions, viz. drought and control. Drought stress was imposed on the rice plants at booting stage by discontinuing watering. The treatment lasted for approximately 7 days until leaf rolling was observed during their heading stage. At that point, mature inflorescences of control and drought-treated pots were collected, snap frozen and kept in -80°C until use. The inflorescence tissues include rachis, branches and spikelets from 3 plants per experimental group. Six small RNA libraries were generated in total for this study that includes two experimental factors: i.e. rice line and condition.

### Preparation of small RNA sequencing library

Total RNA of the inflorescence tissues was isolated using mirVana miRNA Isolation Kit (Invitrogen, Carlsbad, CA) following manufacturer’s instructions. RNA Integrity analysis was performed using Agilent 2100 Bioanalyzer (Agilent Technologies, Santa Clara, CA) where total RNA samples with RIN ≥ 7 was selected for library construction. Total RNA was run in 15% denaturing polyacrylamide gel and small RNAs that lie within 18–30 nt gel fraction were excised and ligated sequentially to 5’ and 3’ proprietary adapters at both termini. Reverse transcription of the small RNA constructs was performed, followed by PCR amplification to produce sequencing libraries. The products were sequenced with Illumina HiSeq System at Beijing Genomics Institute (BGI).

### Bioinformatics analysis workflow

Firstly, quality check of raw reads, which involved filtering of low quality or short reads and adapter trimming, was performed using NGSQC Toolkit v2.3 [41] in order to retrieve clean reads. In mapping and filtering, clean reads were mapped to non-coding RNAs in Sanger Rfam 11.0 (ftp://ftp.ebi.ac.uk/pub/databases/Rfam) [42], repeat sequences in Plant Repeat libraries (http://plantrepeats.plantbiology.msu.edu) [43] and exon and intron of gene models from MSU Rice Genome Annotation Project (ftp://ftp.plantbiology.msu.edu/pub/data/Eukaryotic_Projects/o_sativa/annotation_dbs/pseudomolecules/version_7.0/all.dir/all.gff3) [44]. All the sequence mappings were performed with BLASTn 2.2.27 program [45]. For the identification of known miRNA sequences, clean reads were mapped to *O*. *sativa* miRNA sequences in miRBase version 21 (http://www.mirbase.org) [46] with the following two criteria; (i) reads can be mapped exactly to the miRNA precursors and (ii) reads had a sequence similarity of ≥ 16 nt with mature miRNAs allowing overhangs at both ends. The miRNA expression was expressed in normalized TPM counts before a PCA plot and heatmap were generated using ClustVis [47] to illustrate the variation of miRNA expression among the six samples. |log_2_ fold change| was calculated with normalized TPM counts for the miRNAs between the paired treated and control libraries. miRNAs with |log_2_ fold change| ≥ 1.5 are considered as drought-responsive if their p-values obtained from edgeR [48] are ≤ 0.05. An in-house script was used to extract all the genes within markers RM335 and RM518 (692,760bp-2,026,444bp) of the *qDTY4*.*1* QTL region on chromosome 4 of MSU Rice Genome Annotation Project.

### Prediction of miRNA targets and GO mapping

Sequence complementarity search between identified drought-responsive miRNAs and *O*. *sativa* transcripts (version 7.0) in MSU Rice Genome Annotation was performed with psRNA Target: A Plant Small RNA Target Analysis Server [49] using default setting with a maximum expectation of 3.0. This score depicts the degree of complementarity between a miRNA and its target with higher complementarity giving rise to a smaller value. The predicted targets of selected drought-responsive miRNAs were mapped to GO annotations using agriGO analysis server [50]. Singular Enrichment Analysis in the server was chosen to study the enrichment or over-representation of GO biological processes.

### Quantitative stem-loop RT-PCR for miRNAs

The differential expression of shortlisted drought-responsive miRNAs was validated using a modified protocol of quantitative stem-loop RT-PCR for detection of miRNAs [51]. Firstly, total RNA of the inflorescence tissues was isolated using mirVana miRNA Isolation Kit following manufacturer’s instructions and then treated with RNase-free DNase 1 (Qiagen, Germany). The quality and concentration of the total RNA samples were checked by Nanodrop ND-1000 Spectrophotometer (Thermo Fisher Scientific, USA). In the miRNA-specific reverse transcription protocol, a 20μl reaction mixture contains 1μl total RNA (20ng), 1μl denatured stem-loop RT primer (1μM), 3μl ProtoScript II reaction mix (2X) (New England Biolabs, UK), 0.5μl ProtoScript II enzyme mix (10X) (New England Biolabs, UK) and 14.5μl nuclease-free water. Stem-loop pulsed RT protocol was as follows: 30min at 16°C, followed by pulsed RT of 60 cycles consisting of 30s at 30°C, 30s at 42°C and 1s at 50°C and then incubation at 85°C for 5min to inactivate the reverse transcriptase. As for the 20μl reverse transcription mixture of U6 snRNA reference gene, 1μl random hexamer in the ProtoScript II First Strand cDNA Synthesis Kit (New England Biolabs, UK) was used in place of the 1μl miRNA-specific stem-loop RT primer. Real-time PCR was carried out on Bio-Rad iQ5 (USA) using 5X Hot FIREPol EvaGreen qPCR Mix Plus (no Rox) (Solis BioDyne, Estonia). For miRNA-specific qPCR reaction, 2μl cDNA was mixed with 2μl qPCR Mix Plus (5X) (Solis Biodyne, Estonia), 1μl of forward primer (10μM), 1μl of universal reverse primer (10μM) and 14μl of PCR grade water. For the qPCR of U6 snRNA reference gene, a pair of gene-specific forward and reverse primers of the abovementioned volume and concentration was used instead of the universal reverse primer. Thermocycling conditions for qPCR included: 5min at 95°C, followed by 40 cycles of 15s at 95°C and 1min at 60°C. Verification of the amplification specificity was achieved by generating melting curves. The relative changes in expression of qPCR validated miRNAs were analyzed using the 2^-ΔΔCt^ method [52] with U6 snRNA as reference gene. Primers used in this study are shown in Table 1.

**Table 1:** List of the primers used in quantitative stem-loop RT-PCR for shortlisted drought-responsive miRNAs in the inflorescence tissues of IR64, IR87705-7-15-B and IR77298-14-1-2-10.

Gene	Primer description	Sequence
**osa-miR169d**	Stem-loop primer	GTCGTATCCAGTGCAGGGTCCGAGGTATTCGCACTGGATACGACCCGGCA
	Forward primer	GCGGCGCTAGCCAAGGATGAAT
**osa-miR396c-5p**	Stem-loop primer	GTCGTATCCAGTGCAGGGTCCGAGGTATTCGCACTGGATACGACAAGTTC
	Forward primer	GCGGCGGTTCCACAGCTTTCTT
**osa-miR2275d**	Stem-loop primer	GTCGTATCCAGTGCAGGGTCCGAGGTATTCGCACTGGATACGACTGAGAT
	Forward primer	GGCGGCGGCTTGTTTTTCTCCAAT
**osa-miR5485**	Stem-loop primer	GTCGTATCCAGTGCAGGGTCCGAGGTATTCGCACTGGATACGACTTGCTC
	Forward primer	GCGGCTGACAACTGGTAGCA
**osa-miR5791**	Stem-loop primer	GTCGTATCCAGTGCAGGGTCCGAGGTATTCGCACTGGATACGACCTGGTC
	Forward primer	GCGGCTTGCAGGAGACTAGA
**For all studied miRNAs**	Universal reverse primer	CCAGTGCAGGGTCCGAGGT
**U6 snRNA**	Forward primer	TACAGATAAGATTAGCATGGCCCC
	Reverse primer	GGACCATTTCTCGATTTGTACGTG

## Results and discussion

### An overview of the quality of our small RNA libraries

Inflorescence tissues of IR64, IR87705-7-15-B and IR77298-14-1-2-10 rice lines were harvested under drought-treated and well-watered control conditions at heading stage for high-throughput sequencing. Total RNA integrity (RIN) of the samples were determined using Agilent 2100 Bioanalyzer before constructing the six small RNA sequencing libraries. The RIN values were at least 7.0 in all except for a sample of the IR77298-14-1-2-10 (drought) with RIN of 6.5 (S1 Fig), indicating that intact total RNA samples were used. Following quality control filtering of raw reads, an average of 41612138, 41939724 and 42369350 clean reads was generated for IR64, IR87705-7-15-B and IR77298-14-1-2-10 small RNA libraries respectively (Table 2). Compared to the sequencing output of our previous drought study on vegetative rice [53], the average total clean reads per library in this study (41973737) was ~2.5 times higher than that of 16957778 recorded in our previous study. This is mainly due to the higher throughput per sequencing run provided by the newer version of Illumina HiSeq 2500 Series sequencing system.

**Table 2:** Summary of clean reads and unique reads mapping to different publicly available databases.

Clean reads						
**Categories**	**IR64**		**IR87705-7-15-B**		**IR77298-14-1-2-10**	
	**Well-watered control**	**Drought-treated**	**Well-watered control**	**Drought-treated**	**Well-watered control**	**Drought-treated**
**Total clean reads**	42 085 906	41 138 369	42 187 093	41 692 354	43 604 051	41 134 648
**Exon antisense**	1 136 128 (2.7%)	1 774 124 (4.3%)	2 210 001 (5.2%)	2 106 547 (5.1%)	2 167 204 (5.0%)	2 306 343 (5.6%)
**Exon sense**	6 401 682 (15.2%)	4 503 759 (10.9%)	5 447 522 (12.9%)	7 009 056 (16.8%)	5 069 421 (11.6%)	9 177 961 (22.3%)
**Intron antisense**	1 474 207 (3.5%)	2 112 696 (5.1%)	2 644 178 (6.3%)	2 525 586 (6.1%)	2 634 211 (6.0%)	2 747 751 (6.7%)
**Intron sense**	6 669 641 (15.8%)	4 785 766 (11.6%)	5 776 093 (13.7%)	7 296 210 (17.5%)	5 430 864 (12.5%)	9 490 037 (23.1%)
**Known miRNA**	3 193 764 (7.6%)	2 747 656 (6.7%)	3 545 965 (8.4%)	3 698 852 (8.9%)	3 054 795 (7.0%)	3 185 183 (7.7%)
**rRNA**	1 827 431 (4.3%)	1 516 440 (3.7%)	895 621 (2.1%)	2 346 921 (5.6%)	692 375 (1.6%)	1 183 581 (2.9%)
**Repeat**	2 328 884 (5.5%)	1 949 598 (4.7%)	1 737 196 (4.1%)	2 563 861 (6.1%)	831 674 (1.9%)	903 064 (2.2%)
**snRNA**	54 (0.0%)	39 (0.0%)	39 (0.0%)	52 (0.0%)	30 (0.0%)	47 (0.0%)
**snoRNA**	21 659 (0.1%)	16 920 (0.0%)	20 850 (0.0%)	21 818 (0.1%)	18 127 (0.0%)	22 433 (0.1%)
**tRNA**	921 945 (2.2%)	699 807 (1.7%)	538 006 (1.3%)	1 191 833 (2.9%)	442 420 (1.0%)	682 781 (1.7%)
**Unannotated**	18 110 511 (43.0%)	21 031 564 (51.1%)	19 371 622 (45.9%)	12 931 618 (31.0%)	23 262 930 (53.4%)	11 435 467 (27.8%)
Unique reads						
**Categories**	**IR64**		**IR87705-7-15-B**		**IR77298-14-1-2-10**	
	**Well-watered control**	**Drought-treated**	**Well-watered control**	**Drought-treated**	**Well-watered control**	**Drought-treated**
**Total unique reads**	11 468 394	11 466 281	12 214 795	11 678 019	13 039 986	11 864 885
**Exon antisense**	298 465 (2.6%)	340 184 (3.0%)	323 545 (2.6%)	401 647 (3.4%)	340 879 (2.6%)	354 627 (3.0%)
**Exon sense**	388 507 (3.4%)	428 782 (3.7%)	384 206 (3.1%)	521 252 (4.5%)	410 891 (3.2%)	459 446 (3.9%)
**Intron antisense**	375 649 (3.3%)	426 514 (3.7%)	431 716 (3.5%)	485 814 (4.2%)	469 061 (3.6%)	469 457 (4.0%)
**Intron sense**	460 705 (4.0%)	510 772 (4.5%)	481 624 (3.9%)	594 659 (5.1%)	526 847 (4.0%)	558 583 (4.7%)
**Known miRNA**	1 043 (0.0%)	1 052 (0.0%)	1 075 (0.0%)	1 067 (0.0%)	1 077 (0.0%)	1 066 (0.0%)
**rRNA**	82 409 (0.7%)	71 222 (0.6%)	55 981 (0.5%)	86 685 (0.7%)	65 779 (0.5%)	79 315 (0.7%)
**Repeat**	463 626 (4.0%)	430 799 (3.8%)	471 630 (3.9%)	478 154 (4.1%)	142 711 (1.1%)	133 329 (1.1%)
**snRNA**	15 (0.0%)	8 (0.0%)	14 (0.0%)	20 (0.0%)	14 (0.0%)	19 (0.0%)
**snoRNA**	6 655 (0.1%)	6 066 (0.1%)	6 573 (0.1%)	7 105 (0.1%)	6 413 (0.0%)	7 142 (0.1%)
**tRNA**	16 968 (0.1%)	13 763 (0.1%)	12 579 (0.1%)	17 971 (0.2%)	16 117 (0.1%)	16 175 (0.1%)
**Unannotated**	9 374 352 (81.7%)	9 237 119 (80.6%)	10 045 852 (82.2%)	9 083 645 (77.8%)	11 060 197 (84.8%)	9 785 726 (82.5%)

Percentage of clean and unique reads is indicated in bracket.

**Fig 1:**
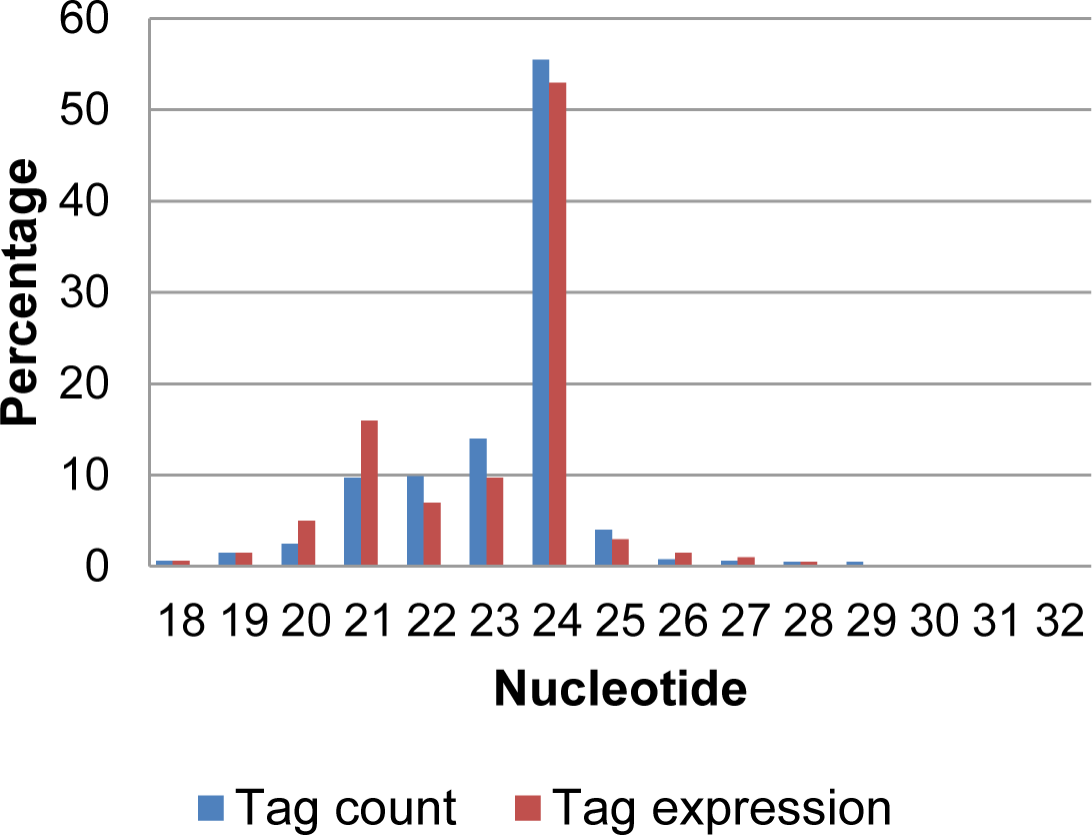
Length distribution of unique small RNA reads. All six generated small RNA libraries displayed two peaks at 21 and 24 nt.

Length distributions of unique reads were then generated for all the six small RNA libraries. All of them consistently displayed two major peaks at 21 and 24 nt, consistent with the size of Dicer-like cleavage product (Fig 1). This may indicate that our libraries are enriched in miRNAs and are suitable for miRNA expression profiling studies. Overall, the mapping results of clean and unique reads to different databases show very consistent proportions (%) among all the libraries (Table 2). Approximately 24% of clean reads per library that mapped to exons, repeats and non-coding RNAs (rRNAs, tRNAs, snRNAs, snoRNAs) were filtered before mapping the remaining small RNA reads to the known rice miRNAs in miRBase 21. Comparatively, Barrera-Figueroa’s research group filtered approximately 27% of clean reads that mapped to rice repeats and the aforementioned non-coding RNAs from each rice inflorescence small RNA library [40].

Datasets from our previous small RNA drought study of the vegetative rice tissues [53] showed that 26.5–44.0% of total clean reads in the small RNA libraries were known rice miRNAs with an average read of 5543518 across the libraries. However, the percentage of known rice miRNA reported in this study was only 7.1–8.6% with an average read of 3237703 across the inflorescence libraries even with higher sequencing throughput. This may imply that the overall endogenous miRNA expression level in rice inflorescence is lower than that in the vegetative tissues. Furthermore, a significantly higher percentage of clean reads mapped to exon and intron of rice gene models in each rice inflorescence library (40.9%) was observed compared to the vegetative library (3.2%). This could be due to secondary small interfering RNAs (siRNAs) that can be generated from protein-coding loci in plant genomes, as first described in *Arabidopsis* [54]. Supporting this notion, two large classes of 21 and 24 nt siRNAs, triggered by osa-miR2118 and osa-miR2275 respectively in their biosynthesis, were reported to show specific spatial expression in rice inflorescence [55,56].

### miRNA expression profile and its annotation

As an important cereal crop and a model species of monocotyledons with complete genome sequence available, rice miRNA has been subjected to an ongoing process of miRNA discovery. Comparative genomic approach between *Arabidopsis* and rice was used in the initial discovery of rice miRNAs, which found 8 conserved miRNAs [57] followed by the identification of 138 miRNAs representing 20 families of rice miRNA genes [58]. Subsequently, an experimental approach that involved cloning and sequencing of rice small RNA cDNA libraries allowed the identification of more highly expressed rice miRNAs [59–61]. The discovery of novel rice miRNA genes is advancing rapidly through the introduction of high-throughput sequencing technology, which allows the detection of lowly expressed miRNAs [17,36]. To date, miRBase (version 21) stores up to 713 mature miRNAs and 592 miRNA precursors for rice.

**Fig 2:**
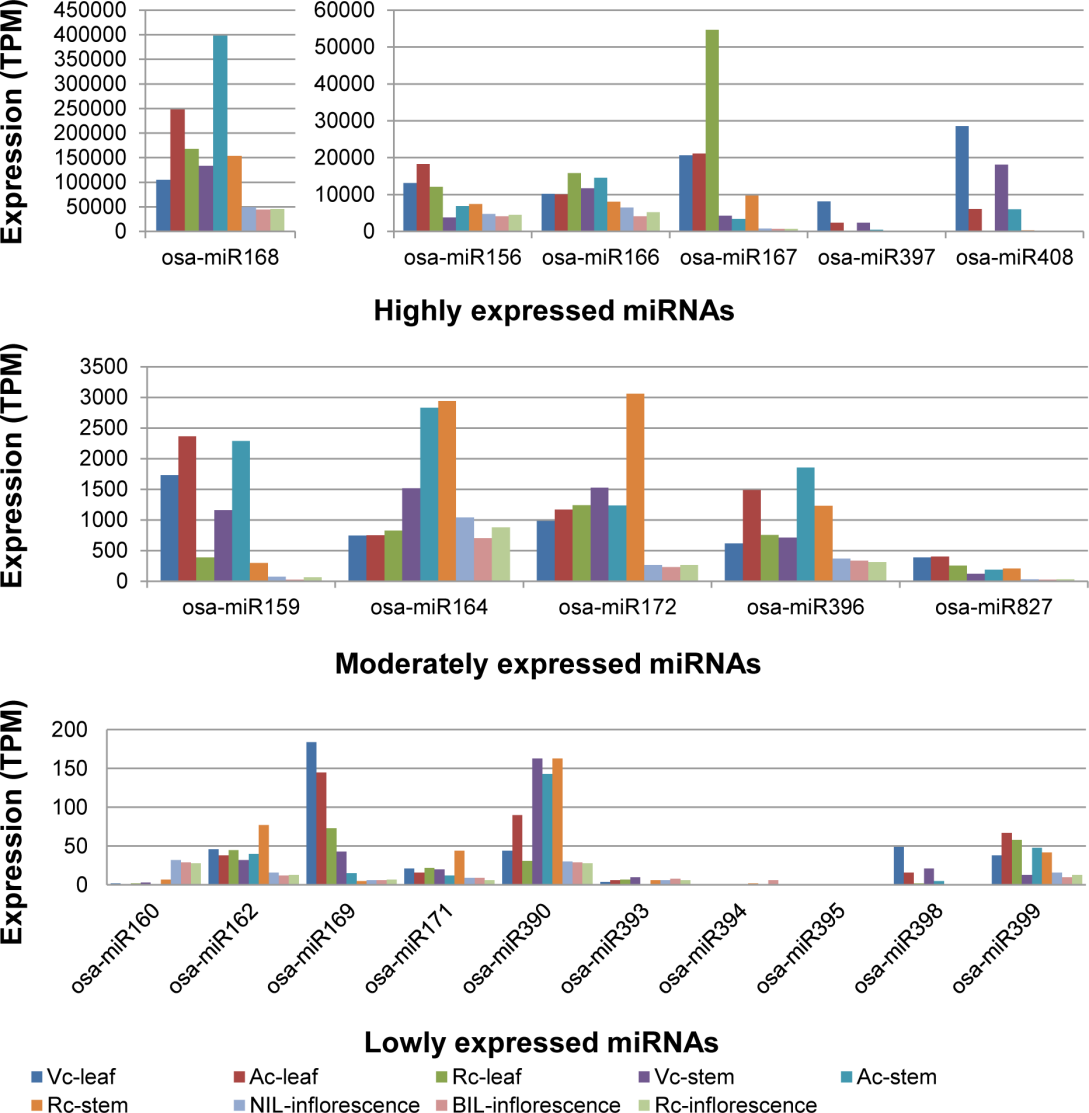
The expression levels of conserved miRNAs in different tissues of studied rice lines under control condition. V = Vandana, A = Aday Sel and R = IR64, NIL = IR87705-7-15-B, BIL = IR77298-14-1-2-10 rice lines while c = control condition. Note: osa-miR167 is moderately expressed while osa-miR159, osa-miR397, osa-miR408 and osa-miR827 are lowly expressed in rice inflorescence. However, due to their higher expression in vegetative tissues, they are shown in the higher expression bar graphs for clearer visualization. osa-miR395 is non-detectable in rice inflorescence.

The expression of 20 conserved miRNA families were detected in our rice inflorescence libraries except osa-miR395 (S1 Table). By identifying the most highly expressed miRNA member from each of the conserved miRNA family and then comparing them among the 20 conserved miRNA families, the conserved miRNA families can be categorized into three groups based on their relative expression levels viz. as high [transcripts per million (TPM)>1000/10000], moderate (TPM = 100–1000) and low (TPM<100) (Fig 2). The classification of conserved miRNAs based on their relative expression levels in rice inflorescence is very similar to that of vegetative tissues found in our previous study [53] (Fig 2). However, our results show that a majority of the conserved miRNAs are expressed at lower levels in rice inflorescence than in leaf and stem tissues as indicated by their lower normalized TPM values in Fig 2. For example, osa-miR159, osa-miR397 and osa-miR408, which were classified under the moderately expressed group in the vegetative tissues, are instead classified under lowly expressed group in the inflorescence tissue. We have previously reported the unique higher expression of osa-miR397, osa-miR408 and osa-miR528 in two of our drought-tolerant rice varieties, especially in the leaf tissues under control condition. They were jointly down-regulated together with osa-miR398 in the leaf tissue of drought-tolerant rice varieties under drought stress as opposed to their up-regulation in susceptible rice variety. These miRNA families commonly target genes encoding copper-containing proteins such as laccases, copper superoxide dismutases, plantacyanin and plastocyanin, which are related to defense and photosynthesis, hence implying their significant post-transcriptional regulatory roles against drought stress in leaf [53].

Non-conserved miRNAs are generally more weakly expressed than conserved miRNAs in the six small RNA libraries, with conserved miRNAs account for 79.3%-89.2% of the reads mapped to known miRNA per library. Out of a total of 183 mature miRNAs from 122 non-conserved families that were identified in the rice inflorescence small RNA libraries (S1 Table), over 156 mature miRNAs had expression level of TPM<10, 19 miRNAs (TPM = 10–100), 7 miRNAs (TPM = 100–1000) and only osa-miR528-5p had TPM>1000. By comparing the non-conserved miRNA expression profiles of rice inflorescence in this study with that of vegetative tissues from our previous study, a greater number of non-conserved miRNAs (316 mature miRNAs) from 184 non-conserved families were found to be expressed in the rice vegetative libraries [53]. Nevertheless, 32 families of non-conserved miRNAs were identified to be expressed in rice inflorescence but not in leaf and stem tissues (bold in S1 Table). Out of the 32 non-conserved miRNA families, 11 families have expression counts >100; (osa-miR2275d, osa-miR5179, osa-miR5486, osa-miR5488, osa-miR5509, osa-miR5793, osa-miR5796, osa-miR5797, osa-miR5800, osa-miR5806, osa-miR5818) and 4 families have expression counts >1000; (osa-miR5485, osa-miR5487, osa-miR5497, osa-miR5791) while the remaining miRNA families have expression count lower than 100. Interestingly, 22 out of the 32 inflorescence-specific non-conserved miRNA families (including all 4 of the families with expression count >1000 above) were induced in the drought-tolerant IR87705-7-15-B rice line under drought stress (bold in S2 Table). This finding draws a strong correlation between inflorescence-specific miRNAs and drought response in rice, which is the primary focus of this experiment.

### Identification of drought-responsive known miRNAs in rice inflorescence

The normalized expression count of each miRNA was compared between drought-treated and control samples of the same rice line in order to identify up- or down-regulated miRNAs. Prior to that, a PCA plot and heatmap were generated with the normalized expression counts to illustrate the variation of miRNA expression among the six small RNA samples (S2 Fig). By referring to the PCA plot (S2 Fig), the first component (PC1) with the highest variance (37.9%) on the X-axis separates the IR87705-7-15-B (drought) sample from its control sample. The second component (PC2) with the second highest variance (23.9%) on the Y-axis separates the IR77298-14-1-2-10 (drought) and IR64 (drought) samples from their respective control samples. This implies that miRNA expression difference between IR87705-7-15-B (control) and IR87705-7-15-B (drought) samples is greater than the paired samples of the other two rice lines.

**Fig 3:**
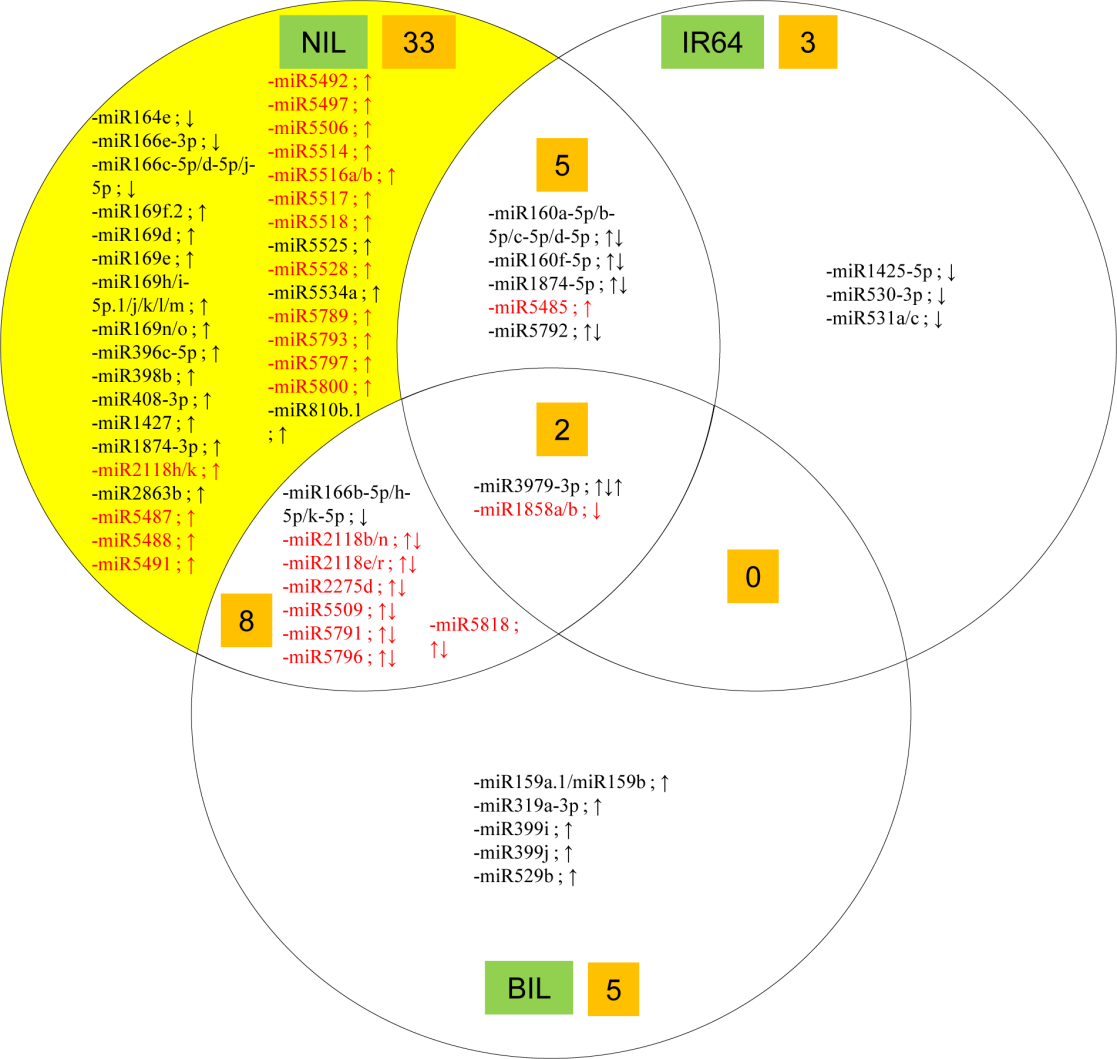
The drought-responsive miRNAs identified in the inflorescence of three rice lines. miRNA names in red are inflorescence-specific non-conserved miRNAs. Differential expression is indicated by arrows in the order of IR87705-7-15-B (NIL), IR64 and IR77298-14-1-2-10 (BIL).

Consistent with the PCA plot’s findings, more drought-responsive known mature miRNAs were identified in the IR87705-7-15-B compared to IR77298-14-1-2-10 and IR64 rice lines (Fig 3). The drought-responsive miRNAs identified in IR87705-7-15-B and IR64 rice lines are very different (Fig 3) despite the tolerant IR87705-7-15-B has high genetic similarity (93.9%) to IR64. This could be explained by the major-effect drought tolerance and yield QTL, *qDTY4*.*1*, which present in tolerant IR87705-7-15-B genome but absent in IR64 genome. Of the 22 inflorescence-specific non-conserved miRNA families that were induced by drought stress in the tolerant IR87705-7-15-B, 6 families were repressed in the IR77298-14-1-2-10, indicating that more commonly identified drought-responsive non-conserved miRNAs were found between the two drought-tolerant rice lines, even though the opposing expression of the 6 miRNA families between IR87705-7-15-B and IR77298-14-1-2-10 remains to be elucidated (highlighted red in Fig 3). The drought-responsive miRNAs identified in inflorescence of the three rice lines are also classified under conserved and non-conserved miRNA families (S2 Table). Overall our results show that there are 9 families of conserved miRNAs and 34 families of non-conserved miRNAs differentially expressed in inflorescence tissue of the three rice lines under drought stress. The higher number of reported drought-responsive non-conserved miRNAs in this study may imply that non-conserved miRNAs play a more prominent role than conserved miRNAs in drought response of rice inflorescence.

For conserved miRNA families, 7 out of 9 of the miRNA families show differential expression in the IR87705-7-15-B except mature miRNAs from osa-MIR159 and osa-MIR399 which were specifically induced in the IR77298-14-1-2-10. It is noteworthy that mature miRNAs from osa-MIR160 were induced in the IR87705-7-15-B while repressed in IR64; and mature miRNAs of osa-MIR166 show down-regulation both in the IR77298-14-1-2-10 and the IR87705-7-15-B. The remaining 5 conserved miRNA families were either specifically repressed (osa-MIR164) or induced (osa-MIR169, osa-MIR396, osa-MIR398 and osa-MIR408) in the IR87705-7-15-B (S2 Table). It is known that miR396 targets growth-regulating factors. The induction of osa-miR396 in IR87705-7-15-B suggests the possibility of repression of growth factors to reserve resources for survival-based processes in the IR87705-7-15-B under drought stress rather than its growth [40]. On the other hand, the over-expression of miR408 enhanced drought tolerance in chickpea by repressing plantacyanin transcript which in turn regulates dehydration-responsive element binding (DREB) genes [62].

Barrera-Figueroa’s group also reported that mature miRNAs from osa-MIR160 and osa-MIR396 are drought induced while osa-MIR166 is drought repressed in rice inflorescence [40]. osa-miR169b and osa-miR169c were also up-regulated by drought stress in their study but the expression fold change was slightly less than 2 [40]. Among the 18 members in osa-MIR169, 11 members show up-regulation in expression in the IR87705-7-15-B of our study. We would like to highlight that the up-regulation of these multiple miRNA members from osa-MIR169 family were not observed in our previous drought study on vegetative tissues, suggesting the potential regulatory importance of this conserved miRNA family in drought response circuitry specifically in rice inflorescence. In addition, miR169 was also reported as up-regulated in roots of wheat [63] and rice [38] under drought stress. Interestingly, MIR169 family had been demonstrated to be involved in the regulation of stress-induced flowering in *Arabidopsis* [64]. The study showed that miR169d over-expressing in transgenic *Arabidopsis* promotes flowering while *AtNF-YA2* (nuclear transcription factor Y subunit, target of miR169d) over-expressing in transgenic *Arabidopsis* delayed flowering. The study also revealed a potential regulatory interaction between miR169/*AtNF-YA* and flowering repressor gene FLOWERING LOCUS C (*FLC*). A repression in *FLC* expression was observed in the miR169d over-expression lines and this in turn induces the expression of FLOWERING LOCUS T (*FT*) and LEAFY (*LFY)*, leading to early flowering. Plants typically exhibit early flowering under drought stress conditions as a drought avoidance mechanism to enhance their chance of surviving through threatening environmental conditions.

For non-conserved miRNA families, 30 out of the identified 34 differentially expressed families were induced in IR87705-7-15-B (S2 Table). Among the 30 up-regulated miRNA families in IR87705-7-15-B, 22 were inflorescence-specific in their expression as they were not reported in the vegetative tissues in our previous study [53] (highlighted red in Fig 3). Their spatial expression in inflorescence tissue was also confirmed with the tissue origin information in miRBase 21. Here, we report for the first time the induction of 23 microRNAs (20 of them are inflorescence-specific in expression—osa-miR5485 until osa-miR5818) (S2 Table) in the IR87705-7-15-B when drought stressed. As the functional information for these non-conserved miRNA families is still limited, our target prediction results show that these miRNA families negatively-regulate a number of important drought-related target genes such as those encoding calmodulin binding protein (osa-miR1427, osa-miR5796), NBS-LRR disease resistance protein (osa-MIR2118), glutathione S-transferase (osa-miR2275d), dehydrin (osa-miR5793), transcription factors and protein kinases (S2 Table).

miR2118 was reported as drought stress induced in *Medicago truncatula* [65] and *Phaseolus vulgaris* [66]. The cleavage of three NBS–LRR protein-encoding transcripts by miR2118 was confirmed by RACE–PCR [67]. Functional analysis by over-expressing miR2118 in tobacco exhibited enhanced drought tolerance via higher water retention potential [68]. osa-miR2275d was also previously revealed to be induced in rice inflorescence under drought stress [40] and it was predicted to target glutathione S-transferase. Glutathione S-transferase was proposed to be a negative component of stress-mediated signal transduction pathways as knockout *Arabidopsis* transgenic lines were more tolerant to drought and salt stresses by accumulating higher levels of glutathione and abscisic acid [69]. In addition, osa-miR2118 and osa-miR2275 have been reported to target numerous genomic clusters in rice genome to produce 21 and 24 nt siRNAs in a phased manner specifically in rice inflorescence [55,56]. Although the information on the biogenesis or the functions of siRNAs is still lacking in rice, they were generally known to be involved in guiding DNA methylation for epigenetic modification or DNA methylation of protein-coding gene promoters that leads to specific gene silencing. In fact, recent experimental evidence has pointed out that siRNAs are crucial for control of pollen fertility in rice [70,71]. Additionally, *sac9* (target gene of osa-miR5792) mutants expressed a systemic stressed phenotype including over-expression of stress-induced genes, accumulation of reactive oxygen species, closed guard cell, dwarfed and slow-growing phenotype [72].

Furthermore, three of our predicted target genes have inflorescence-specific functions. They are genes encoding S-locus-like receptor protein kinase (osa-miR5485), spotted leaf 11 (osa-miR5492) and KH domain containing protein (osa-miR5517). S-locus-like receptor was first discovered as a stigma-specific plasma membrane-localized transmembrane protein kinase with self-incompatibility function to limit self-fertilization in *Brassica* [73]. Since rice is self-fertilising, this protein kinase may have other functions besides reproductive recognition. There is evidence that shows it is responsive to environmental stresses, i.e. low temperatures, short-day photoperiod or water limitation [74]. On the other hand, spotted leaf 11 (E3 ubiquitin ligase) controls flowering time via its interaction with SPIN1, a KH domain protein where mutation of spotted leaf 11 gene and over-expression of *SPIN1* were respectively found to delay rice flowering [75].

### Functional annotation of differentially expressed genes in the IR87705-7-15-B

**Fig 4:**
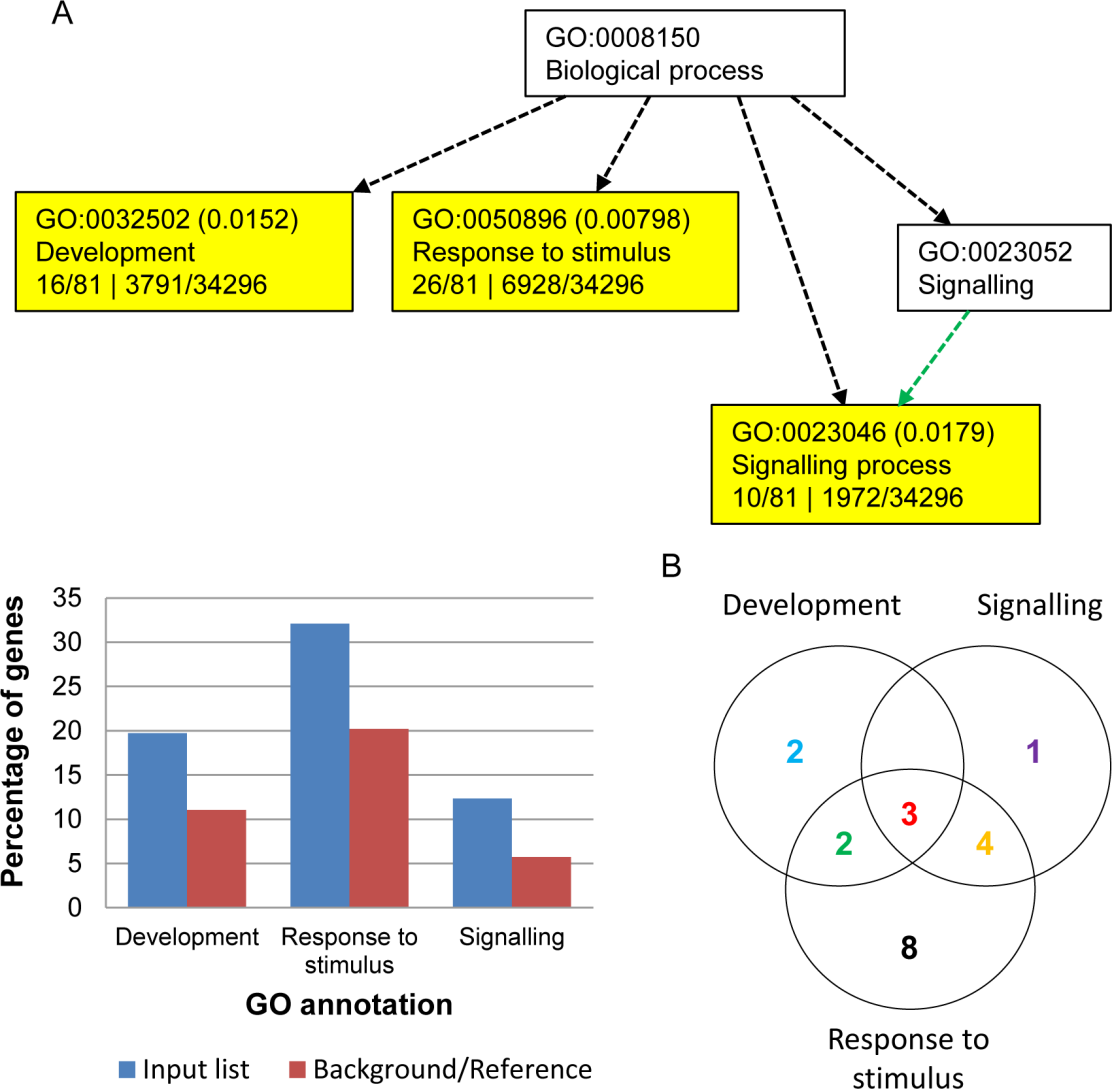
GO annotation of the target genes of drought-responsive miRNAs in IR87705-7-15-B. (A) The hierarchical graph and bar chart shows the over-representation of development, response to stimulus and signalling processes when AGRIGO Singular Enrichment Analysis (SEA) was carried out on 94 predicted targets of 27 miRNAs. (B) The Venn diagram displays the numerical distribution of miRNAs with targets annotated to at least 1 of the over-represented biological processes.

**Fig 5:**
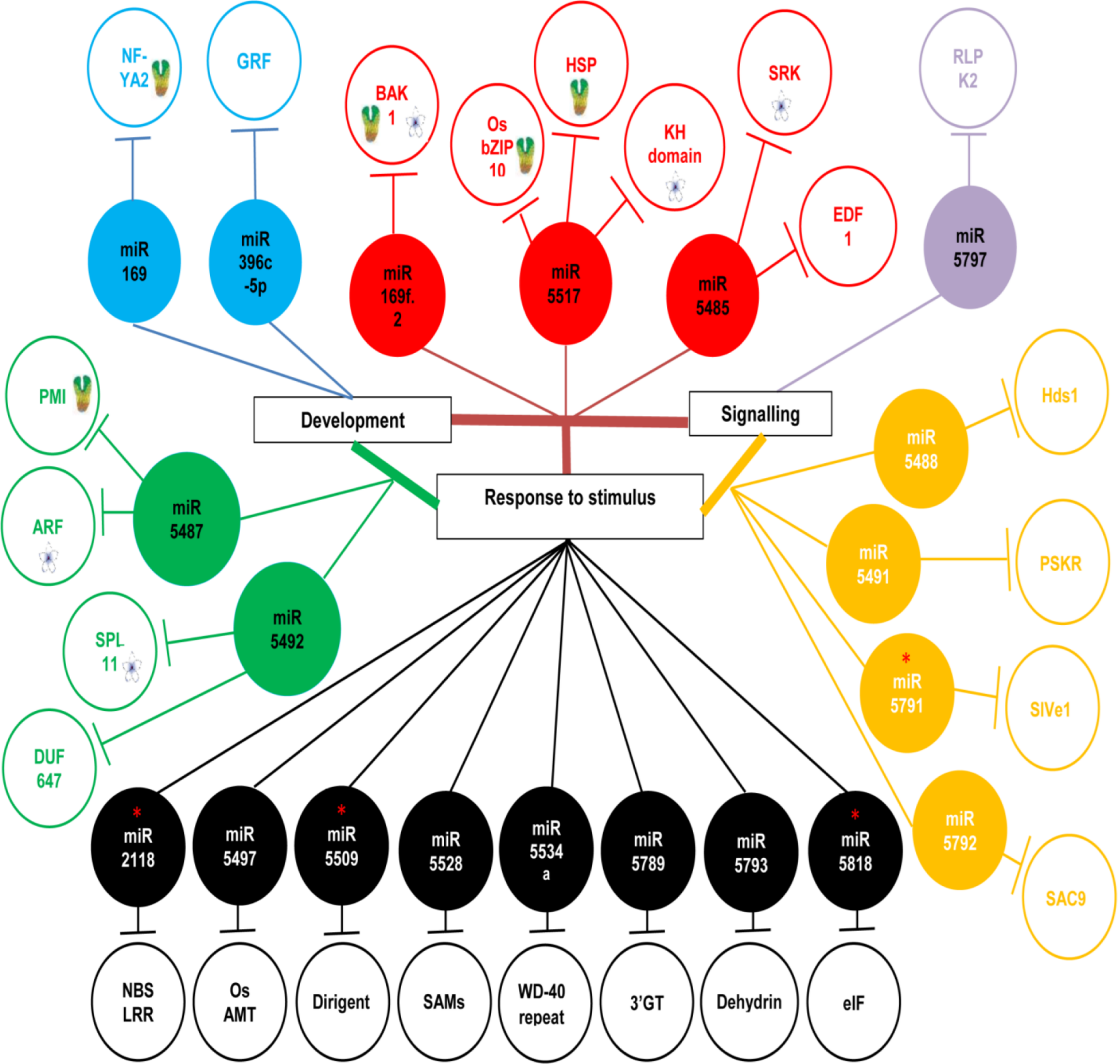
20 drought-induced miRNAs in IR87705-7-15-B with their corresponding target genes annotated to three GO processes. The coloration of each subgroup is in accordance to Fig 4B. The red asterisks indicate the 4 inflorescence-specific non-conserved miRNAs that were repressed in IR77298-14-1-2-10 while induced in IR87705-7-15-B under drought stress. Refer S3 Table for detailed GO annotation report. NFYA2 = nuclear transcription factor Y subunit, GRF = growth regulating factor, BAK1 = BRASSINOSTEROID INSENSITIVE 1-associated receptor kinase 1 precursor, OsbZIP10 = bZIP transcription factor, HSP = heat shock cognate 70 kDa protein 2, KH domain = KH domain containing protein, SRK = S-locus-like receptor protein kinase, EDF = endothelial differentiation-related factor 1, RLPK2 = receptor-like protein kinase 2 precursor, PMI = mannose-6-phosphate isomerase, ARF = auxin response factor, SPL11 = spotted leaf 11, DUF647 = DUF647 domain containing protein, NBS-LRR = NBS-LRR disease resistance protein, OsAMT = ammonium transporter protein, SAMs = S-adenosylmethionine synthetase, WD-40 repeat = katanin p80 WD40 repeat-containing subunit B1 homolog 1, 3’GT = anthocyanin 3-O-beta-glucosyltransferase, eIF = eukaryotic translation initiation factor, Hds1 = hydroxymethylbutenyl 4-diphosphate synthase, PSKR = phytosulfokine receptor precursor, SlVe1 = resistance protein SlVe1 precursor.

To gain a better insight into the regulatory roles of these little known inflorescence-specific non-conserved miRNAs, which are induced in the IR87705-7-15-B under drought stress (highlighted red in Fig 3), Gene Ontology (GO) annotation analysis was performed on their predicted targets together with that of other miRNAs that were specifically differentially expressed in the IR87705-7-15-B (yellow space in Fig 3). As 94 predicted targets of 27 miRNAs were inputted into the analysis, the results show over-representation of three biological processes, namely development (GO:0032502), response to stimulus (GO:0050896) and signalling (GO:0023046) (Fig 4A). Fig 4B shows the distribution of 20 miRNAs with annotated targets across the three biological processes and the detailed information of these 20 miRNAs including miRNA name, target name and corresponding GO information are shown in (Fig 5, S3 Table).

Interestingly, our results show that four inflorescence-specific miRNAs viz. osa-miR5485, osa-miR5487, osa-miR5492 and osa-miR5517 together with two non-inflorescence specific miRNAs viz. osa-miR169d and osa-miR169f.2 targets genes that are involved specifically in flower or embryonic development (Fig 5, S3 Table). Among them, osa-miR169f.2, osa-miR5517 and osa-miR5485 regulate target genes that encompass all the three aforementioned biological processes and thus may point us to their crucial roles in the regulatory network of drought response. Firstly, osa-miR169f.2 was for the first time reported in this study to target the transcript of BRASSINOSTEROID INSENSITIVE 1-associated receptor kinase 1 precursor (*BAK1*). In brassinosteroid signalling, the interaction of BAK1 with BRASSINOSTEROID INSENSITIVE 1 (BRI1) is crucial for the perception of brassinosteroid at the plasma membrane [76]. Brassinosteroids consist of a class of essential phytohormones that are involved in monitoring a plethora of plant growth and developmental processes including seed germination, stem and root elongation, senescence, vascular differentiation, reproduction and photomorphogenesis. Besides regulating multiple physiological functions, they also respond to various biotic and abiotic stresses [77,78].

Next, three predicted targets of osa-miR5517 were found to be very much related to molecular drought regulation, viz. OsbZIP10 transcription factor, heat shock cognate 70kDa protein 2 and a KH domain containing protein. There are 89 OsbZIP transcription factor-encoding genes in the rice genome and 26 *OsbZIP* genes were up-regulated and 11 *OsbZIP* genes were down-regulated under one or more of the dehydration, cold and high salinity stress treatments. Among them, *OsbZIP10* was reported as induced under dehydration stress and is associated with pollination/fertilisation and/or seed development [79]. Heat shock proteins, also accumulated in response to dehydration stress, are a class of molecular chaperones that assist in cellular protein folding, protein transport across membranes and protein degradation [80]. KH domain-containing putative RNA-binding protein was reported as an upstream negative gene regulator critical for heat stress response where its mutant *Arabidopsis* lines displayed enhanced tolerance to heat stress compared to wild-type [81]. Besides that, in our previous section, we have discussed the possible involvement of osa-miR5517 (KH domain containing protein), osa-miR5492 (spotted leaf 11) and osa-miR169d (nuclear transcription factor Y subunit) in controlling flowering time while osa-miR5485 (S-locus-like receptor protein kinase) may have functions that extend beyond self-incompatibility.

In this GO annotation analysis, 8 miRNAs were reported to specifically target genes involved in response to stimulus process (GO:0050896) (Fig 5, S3 Table). While the potential role of 2 miRNAs and their corresponding targets (osa-miR2118, osa-miR5793) in drought stress regulation has been discussed in the previous section, the functions of the predicted targets of the remaining 6 miRNAs are also very much relevant to drought response in rice. For instance, osa-miR5497 was predicted to target *OsAMT2*.*3*, one of the twelve reported ammonium transporter membrane proteins in rice genome which is integral for ammonium ion uptake as a preferred nitrogen source [82]. Nitrogen is an important nutrient for plant growth and development, which is a process markedly reduced by drought stress. Further, dirigent protein (target of osa-miR5509) is known to play an important role in lignin biosynthesis. An accumulation of its activity was found in resurrection plant *Boea hygrometrica* under drought stress and was suggested to alter the mechanical strength and flexibility of plant cell wall in favor of plant recovery following rewatering [83]. Likewise, S-adenosylmethionine synthetase (SAMs; target of osa-miR5528) displayed an increased activity under drought stress while decreased under flooding stress in soybean [84]. SAMs catalyzes biosynthesis of S-adenosylmethionine (SAM) from methionine and ATP. SAM in turn is involved in the metabolism of pectin, lignin and ethylene in plants. Ethylene is an important plant hormone that functions in a range of physiological processes such as fruit ripening, flowering, leaf abscission and stress responses. Thus, it can be deduced that SAMs is crucial for growth and development of plants [85]. In addition, eukaryotic translation initiation factors (eIF; target of osa-miR5818), which act as a rate-limiting step in translation or protein synthesis, have been ascribed a role in regulating developmental processes such as pollen germination and embryogenesis [86]. *eIF*-overexpressing *Arabidopsis* displayed significantly higher survival rate, soluble proteins, photosynthetic efficiency and enhanced photooxidative tolerance under drought conditions [87].

**Fig 6:**
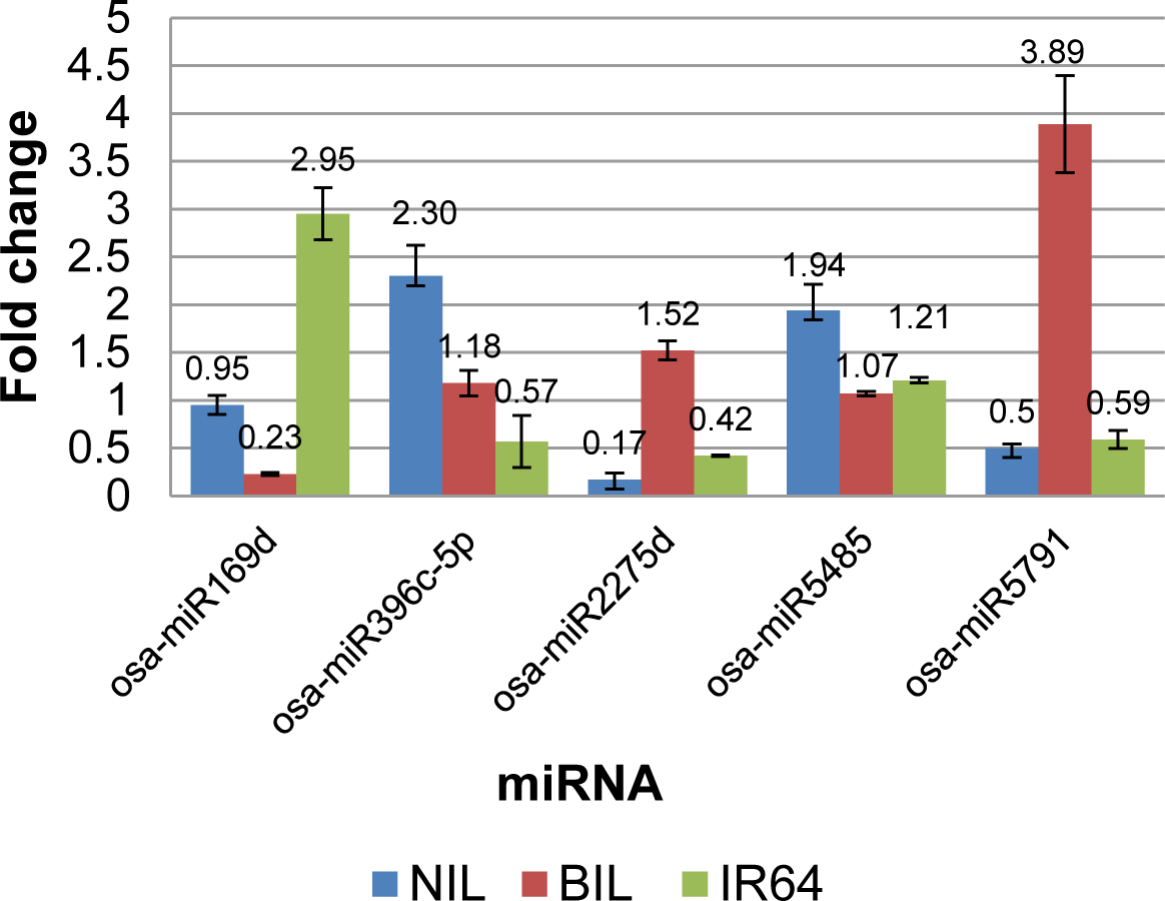
Fold change of quantitative stem-loop RT-PCR for shortlisted miRNAs. The height of bar represents mean of normalized fold change in triplicates and its corresponding standard deviation is represented by error bar. NIL = IR87705-7-15-B and BIL = IR77298-14-1-2-10.

Using quantitative stem-loop RT-PCR, the differential expression of 5 shortlisted miRNAs, of which 3 are inflorescence-specific non-conserved miRNAs, were validated (Fig 6). Consistent with Illumina sequencing results, osa-miR396c-5p and osa-miR5485 were validated by qPCR as induced in the IR87705-7-15-B inflorescence under drought stress. osa-miR2275d and osa-miR5791, which were shown by Illumina sequencing result as induced in the IR87705-7-15-B but repressed in the IR77298-14-1-2-10 inflorescence under drought stress, were otherwise repressed in the IR87705-7-15-B but induced in the IR77298-14-1-2-10 according to our qPCR result. Further, qPCR results show that osa-miR169d was up-regulated in IR64 as opposed to its up-regulation in IR87705-7-15-B as revealed by Illumina sequencing. However, based on the observation of multiple miRNA members from osa-MIR169 (i.e. 11 out of 18 miRNA members) being induced in IR87705-7-15-B under drought via Illumina sequencing, osa-miR169 is very likely to play a significant role in drought response mechanisms of IR87705-7-15-B. The disparity of results between Illumina sequencing and qPCR was also observed in our previous drought study on vegetative rice and this scenario could be caused by: (i) difference in fresh plant tissues for qPCR analysis, (ii) although the drought stress treatments in greenhouse were identical, the expression of miRNAs may fluctuate as determination of tissue sampling time based on the leaf rolling is not precise enough [53]. For example, a miRNA i.e. miR398 was reported to exhibit dynamic expression in poplars where it was initially up-regulated at 3–4 h of ABA or NaCl stress but was then down-regulated after 48 h and eventually increased again after 72 h [88].

Taken together, our qPCR findings still demonstrates that these miRNAs are drought-responsive in rice inflorescence, however the discrepancy of change in miRNA expression observed between Illumina sequencing and qPCR results necessitate further expression and functional characterization experiments to be conducted on the reported drought-responsive miRNAs of this study. Recently, the discovery of endogenous target mimicry (eTM) [89] and the establishment of CRISPR/Cas9 system [90] in rice have enabled functional studies of drought-responsive miRNA(s) and their target genes to be designed and implemented in an easier, more efficient and less expensive manner. CRISPR/Cas9 is capable of removing/inserting/replacing multiple genes at the same time and this system is feasible in different plant species. This emerging technology will certainly pave the way for the advancement of miRNA-based molecular breeding to improve the drought tolerance and yield of rice plants under drought stress.

### Identification of candidate genes in *qDTY4*.*1* QTL

A total of 199 genes were found in the *qDTY4*.*1* QTL region which is between markers RM335-RM518 (692,760bp-2,026,444bp) on chromosome 4 (S4 Table). It is noteworthy that the drought-responsive miRNA genes reported in this study were not found in this QTL region but were localized in different chromosomes instead. This observation can be explained by the trans-acting nature of miRNAs in gene regulation where a miRNA gene codes for a miRNA that will then regulate the expression of another target gene. Although a majority (146/199) of the genes encode for retrotransposons, transposon, unknown expressed or hypothetical proteins, two groups of genes; NBS-LRR resistance proteins (7 copies) and subtilisin (11 copies) were observed to exist in multiple copies in this region.

Plant resistance genes (R genes) exist in large families and typically consist of a nucleotide-binding site domain and a leucine-rich repeat domain, represented by NBS-LRR. Although NBS-LRR proteins are often implicated in protein-protein interaction with pathogen-derived molecules to trigger plant defense, there are studies that demonstrate that miR2118, which guides the mRNA cleavage of NBS-LRR resistance genes, is closely related to drought response in legumes [65,66,91]. While our target prediction analysis did not show any direct evidence of osa-miR2118 targeting the NBS-LRR resistance genes in *qDTY4*.*1*, four NBS-LRR resistance genes, namely LOC_04g10380.1, LOC_04g30610.1, LOC_04g30660.1 and LOC_04g30690.1, were targeted on chromosome 4. Further experimental validation of miRNA:mRNA interaction is required to examine whether osa-miR2118 is directly involved in the post-transcriptional regulation of NBS-LRR resistance genes in *qDTY4*.*1*. Subtilisin is a family of serine proteases that catalyzes hydrolysis of peptide bonds. Breaking down of non-functional and potentially harmful protein is vital for the adaptation and survival of plants to dehydration stress [92].

A gene encoding for potassium channel KAT2 (1 copy) was identified within this QTL region. Potassium channel KAT2 is expressed in guard cells and functions to regulate stomatal movement through the transport of K^+^ which in turn results in turgor change of the two guard cells surrounding the stoma. Stomatal closure is an important plant adaptation to fluctuating or stressing natural environments [93]. Genes encoding for ABA biosynthesis and catabolism were also found within this QTL region. They are epoxycarotenoid dioxygenase (*NCED*) (1 copy) and cytochrome P450 (2 copies). ABA is best known as a plant hormone and signalling molecule where ABA-mediated signalling pathway is indispensable for adaptive stress processes in plants [94]. The precise balance between its biosynthesis and catabolism controls the hormonal action of ABA in plants. NCED is the key ABA biosynthesis enzyme as it catalyzes the regulating steps of the biosynthetic process [95]. A total of 5 *NCED* genes have been discovered so far in *Arabidopsis* and rice. Conversely, cytochrome P450 was reported to play a predominant role in ABA catabolism through 8’-hydroxylation of ABA to phaseic acid [96].

Another gene encoding for 3-ketoacyl-CoA synthase 6 (1 copy) was identified in this QTL region. This protein is essential for cuticular wax biosynthesis through both decarbonylation and acyl-reduction wax synthesis pathways. Cuticle is a waxy protective layer covering the epidermis of aerial plant organs. It functions to reduce non-stomatal water loss besides being a physical barrier against pathogens and UV radiation [97]. Along this line, a functional study of *OsGL1-6* gene, a member of the fatty aldehyde decarbonylase gene family, indicated its role in the build-up of leaf cuticular wax that is responsible for increased drought resistance in rice [98].

## Conclusions

Six miRNA libraries from the inflorescence of three rice lines with contrasting levels of drought tolerance were generated under control and drought stress conditions. Selection of the drought-tolerant IR87705-7-15-B with high genetic similarity to the drought-susceptible IR64 enabled us to better identify the drought-responsive miRNAs by reducing the false positives detected due to use of heterogeneous genetic stocks. The length distributions of unique reads generated for the six small RNA libraries showed two major peaks at 21 and 24 nt, indicating that the small RNA libraries are enriched in miRNAs. Although the classification of conserved miRNA families based on their relative expression levels in rice inflorescence is comparable to that in the vegetative tissues of leaf and stem found in our previous study, majority of the conserved miRNAs are expressed at lower levels in rice inflorescence than in leaf and stem tissues. 32 families of non-conserved miRNAs were observed to be specifically expressed in rice inflorescence of which 22 families were up-regulated in the drought-tolerant IR87705-7-15-B. Overall 9 conserved and 34 non-conserved miRNA families were identified as drought-responsive in rice inflorescence with 5 conserved and 30 non-conserved families induced in the IR87705-7-15-B respectively. The higher number of identified drought-responsive non-conserved miRNAs may imply that non-conserved miRNAs has a more prominent role to play than conserved miRNAs in drought response of rice inflorescence. GO annotation of the target genes of drought-responsive miRNAs identified in the IR87705-7-15-B revealed over-representation of three biological processes, including development, response to stimulus and signalling. Interestingly, our results show that four inflorescence-specific miRNAs viz. osa-miR5485, osa-miR5487, osa-miR5492 and osa-miR5517 together with two non-inflorescence specific miRNAs viz. osa-miR169d and osa-miR169f.2 target genes that are involved specifically in flower or embryonic development. Among these miRNAs, osa-miR169d, osa-miR5492 and osa-miR5517 are related to flowering time control. It is noteworthy that osa-miR2118 and osa-miR2275, which function in the biosynthesis of rice inflorescence-specific 21 and 24 nt siRNAs, were induced in the IR87705-7-15-B but repressed in the IR77298-14-1-2-10. In addition, gene searching in *qDTY4*.*1* QTL region revealed multiple copies of NBS-LRR resistance genes and subtilisin together with genes implicated in stomatal movement, ABA metabolism and cuticular wax biosynthesis. Further dissection of the inflorescence-specific non-conserved miRNAs induced under the drought stress in IR87705-7-15-B is required to assign precise biological functions at the molecular level for drought response.

## Supporting information

S1 Table The Illumina sequencing expression profile of known *Oryza sativa* mature miRNAs deposited in miRBase 21. ^$^ Mature miRNA are recorded together in the same cell with // as separator because they have identical mature miRNA sequence. ^$^ Some non-conserved mature miRNAs are bolded because they were detected in the inflorescence tissue of this study but were not detected in rice vegetative tissues of our previous study [53]. ^¥£#^ These columns show the expression level counts in all 6 libraries. ^¥£#^ The expression level counts of some mature miRNAs are left blank because they were not detected in the inflorescence tissue of this study but were detected in rice vegetative tissues of our previous study [53].
(XLSX)

S2 Table Drought-responsive miRNAs identified in the inflorescence of 3 studied rice lines. 22 families of inflorescence-specific non-conserved miRNA families are in bold. MSU identifiers of predicted gene targets that are annotated in our Gene Ontology analysis are indicated above. Up and down regulated miRNAs indicated.
(DOCX)

S3 Table Detailed GO annotation of the target genes of 20 drought-induced miRNAs in IR87705-7-15-B annotated to development, response to stimulus and signalling processes. (DOCX)

S4 Table Identification of candidate genes in qDTY4.1 QTL. (XLSX)

S1 Fig RNA quality: RNA integrity (RIN) analysis. IR = IR64, OFF = NIL (IR87705-7-15-B) and P1 = BIL (IR77298-14-1-2-10) rice lines; C = control and D = drought-treated conditions.
(DOCX)

S2 Fig PCA plot and heatmap was generated with normalized expression of miRNAs from the six samples. NIL = IR87705-7-15-B and BIL = IR77298-14-1-2-10 rice lines.
(DOCX)

## Abbreviations

IRRI: International Rice Research Institute
BIL: backcross inbred line
QTL: quantitative trait locus
NIL: near isogenic line
miRNA: microRNA
nt: nucleotide
mRNA: messenger RNAs
AGO: Argonaute
AP2: APETALA
SPLs: SQUAMOSA promoter binding protein-like
RIN: RNA integrity number
siRNA: small interfering RNA
TPM: transcripts per million
NF-YA2: nuclear transcription factor Y subunit
GO: Gene Ontology
BAK1: BRASSINOSTEROID INSENSITIVE 1-associated receptor kinase 1 precursor
NCED: epoxycarotenoid dioxygenase
