# Supplementary material for: Identification of functionally important microRNAs from rice inflorescence at heading stage of a qDTY4.1-QTL bearing Near Isogenic Line under drought conditions
Source: PLoS One. 2017 Oct 18;12(10):e0186382. doi: 10.1371/journal.pone.0186382 (PMC5647096; doi:10.1371/journal.pone.0186382)
Supplement: S2 Table — 22 families of inflorescence-specific non-conserved miRNA families are in bold. MSU identifiers of predicted gene targets that are annotated in our Gene Ontology analysis are indicated above. Up and down regulated miRNAs indicated. (DOCX) [file pone.0186382.s002.docx]

**S2 Table**

**Drought-responsive miRNAs identified in the inflorescence of 3 studied rice lines.**

| **No** | **MicroRNAs** | **IR64** | **IR87705-7-15-B** | **IR77298-14-1-2-10** | **Predicted targets** |
| --- | --- | --- | --- | --- | --- |
| **Conserved microRNAs** | | | | | |
| 1 | osa-miR159a.1//osa-miR159b |  |  | 1215-4082 ↑ | MYB family transcription factor |
|  | osa-miR319a-3p |  |  | 186-716 ↑ |  |
| 2 | osa-miR160a-5p to osa-miR160d-5p | 286-44↓ | 92-283↑ |  | Auxin response factor |
|  | osa-miR160f-5p | 13-5↓ | 3-38↑ |  |  |
| 3 | osa-miR164e |  | 2890-58 ↓ |  | NAC transcription factor |
|  | osa-miR166e-3p |  | 138-33 ↓ |  | Alkaline neutral invertase |
| 4 | osa-miR166b-5p// osa-miR166h-5p// osa-miR166k-5p |  | 448-148 ↓ | 300-64 ↓ | Exonuclease, 1,3-beta-glucan synthase component domain containing protein |
|  | osa-miR166c-5p// **osa**-miR166d-5p// osa-miR166j-5p |  | 702-247 ↓ |  | Exonuclease |
| 5 | osa-miR169f.2 |  | 48-1108 ↑ |  | BRASSINOSTEROID INSENSITIVE 1-associated receptor kinase 1 precursor (LOC_Os11g31560.1) |
|  | osa-miR169d |  | 9-48 ↑ |  | Nuclear transcription factor Y subunit (LOC_Os03g48970.1) |
|  | osa-miR169e |  | 38-118 ↑ |  | Nuclear transcription factor Y subunit |
|  | osa-miR169h//osa-miR169i-5p.1//osa-miR169j//osa-miR169k//osa-miR169l//osa-miR169m |  | 12-45 ↑ |  | Nuclear transcription factor Y subunit |
|  | osa-miR169n//osa-miR169o |  | 8-32 ↑ |  | Nuclear transcription factor Y subunit |
| 6 | osa-miR396c-5p |  | 218-693 ↑ |  | Growth-regulating factor |
| 7 | osa-miR398b |  | 9-37 ↑ |  | Copper/Zinc superoxide dismutase |
| 8 | osa-miR399i |  |  | 21-87 ↑ |  |
|  | osa-miR399j |  |  | 53-169 ↑ |  |
| 9 | osa-miR408-3p |  | 151-590 ↑ |  | Plastocyanin-like domain containing protein |
| **Non-conserved microRNAs** | | | | | |
| 1 | osa-miR1425-5p | 686-174 ↓ |  |  | Rf1, mitochondrial precursor |
| 2 | osa-miR1427 |  | 125-360 ↑ |  | Calmodulin binding protein |
| 3 | osa-miR1874-3p |  | 120-698 ↑ |  |  |
|  | osa-miR1874-5p | 127-43 ↓ | 6-91 ↑ |  |  |
| 4 | **osa-miR2118b// osa-miR2118n** |  | 1-36 ↑ | 11-1 ↓ |  |
|  | **osa-miR2118e// osa-miR2118r** |  | 1-5 ↑ | 6-1 ↓ | NBS-LRR disease resistance protein |
|  | **osa-miR2118h// osa-miR2118k** |  | 1-6 ↑ |  |  |
| 5 | **osa-miR2275d** |  | 24-373 ↑ | 138-2 ↓ | Glutathione S-transferase |
| 6 | osa-miR2863b |  | 2-22 ↑ |  |  |
| 7 | osa-miR3979-3p | 14-3 ↓ | 2-16 ↑ | 1-4 ↑ | Translation elongation factor protein |
| 8 | osa-miR529b |  |  | 166-498 ↑ | SBP-box gene family |
| 9 | osa-miR530-3p | 7523-2148 ↓ |  |  | Golgi snare 12 protein |
| 10 | osa-miR531a// osa-miR531c | 208-13 ↓ |  |  | Valine-pyruvate aminotransferase 3 |
| 11 | **osa-miR5485** | 217-786 ↑ | 325-6387 ↑ |  | S-locus-like receptor protein kinase (LOC_Os03g30890.1),  Endothelial differentiation-related factor 1 (LOC_Os06g39240.1) |
| 12 | **osa-miR5487** |  | 451-1462 ↑ |  | Mannose-6-phosphate isomerase (LOC_Os11g38810.1),  Auxin response factor (LOC_Os04g57610.1) |
| 13 | **osa-miR5488** |  | 7-105 ↑ |  | Hydroxymethylbutenyl 4-diphosphate synthase (LOC_Os02g39160.1) |
| 14 | **osa-miR5491** |  | 25-67 ↑ |  | Phytosulfokine receptor precursor (LOC_Os02g05950.1) |
| 15 | **osa-miR5492** |  | 7-26 ↑ |  | Spotted leaf 11 (LOC_Os07g39590.1), DUF647 domain containing protein (LOC_Os04g43690.1) |
| 16 | **osa-miR5497** |  | 299-1188 ↑ |  | Ammonium transporter protein (LOC_Os01g61550.1) |
| 17 | **osa-miR5506** |  | 20-88 ↑ |  |  |
| 18 | **osa-miR5509** |  | 1-116 ↑ | 24-1 ↓ | Dirigent (LOC_Os10g18870.1) |
| 19 | **osa-miR5514** |  | 2-13 ↑ |  |  |
| 20 | **osa-miR5516a// osa-miR5516b** |  | 4-14 ↑ |  |  |
| 21 | **osa-miR5517** |  | 0-13 ↑ |  | bZIP transcription factor (LOC_Os01g64000.1), Heat shock cognate 70 kDa protein 2 (LOC_Os12g38180.1), KH domain containing protein (LOC_Os12g40560.1) |
| 22 | **osa-miR5518** |  | 4-27 ↑ |  |  |
| 23 | osa-miR5525 |  | 103-490 ↑ |  | OsWAK128b - OsWAK receptor-like protein kinase |
| 24 | **osa-miR5528** |  | 5-17 ↑ |  | S-adenosylmethionine synthetase (LOC_Os01g18860.1) |
| 25 | osa-miR5534a |  | 1-75 ↑ |  | Katanin p80 WD40 repeat-containing subunit B1 homolog 1 (LOC_Os01g57210.1) |
| 26 | **osa-miR5789** |  | 2-41 ↑ |  | Anthocyanin 3-O-beta-glucosyltransferase (LOC_Os01g45140.1) |
| 27 | **osa-miR5791** |  | 73-2605 ↑ | 738-60 ↓ | Resistance protein SlVe1 &SlVe2 precursor |
| 28 | osa-miR5792 | 43-10 ↓ | 8-58 ↑ |  | SAC9 (LOC_Os01g25330.1) |
| 29 | **osa-miR5793** |  | 73-260 ↑ |  | Dehydrin (LOC_Os11g26780.1) |
| 30 | **osa-miR5796** |  | 3-118 ↑ | 28-5 ↓ | Calmodulin binding protein |
| 31 | **osa-miR5797** |  | 14-125 ↑ |  | Receptor-like protein kinase 2 precursor (LOC_Os11g36140.1) |
| 32 | **osa-miR5800** |  | 57-175 ↑ |  |  |
| 33 | **osa-miR5818** |  | 11-241 ↑ | 46-7 ↓ | Eukaryotic translation initiation factor (LOC_Os01g73880.1) |
| 34 | osa-miR810b.1 |  | 17-88 ↑ |  |  |

22 families of inflorescence-specific non-conserved miRNA families are in bold. MSU identifiers of predicted gene targets that are annotated in our Gene Ontology analysis are indicated above. Up and down regulated miRNAs indicated.
