# Supplementary material for: Identification of functionally important microRNAs from rice inflorescence at heading stage of a qDTY4.1-QTL bearing Near Isogenic Line under drought conditions
Source: PLoS One. 2017 Oct 18;12(10):e0186382. doi: 10.1371/journal.pone.0186382 (PMC5647096; doi:10.1371/journal.pone.0186382)
Supplement: S3 Table — (DOCX) [file pone.0186382.s003.docx]

**S3 Table**

**Detailed GO annotation of the target genes of 20 drought-induced miRNAs in IR87705-7-15-B annotated to development, response to stimulus and signalling processes.**

| **Categories** | **Mature miRNA** | **Target(s)** | **GO annotation** |
| --- | --- | --- | --- |
| Development | osa-miR169d  osa-miR396c-5p | Nuclear transcription factor Y subunit  (LOC_ Os03g48970.1)  Growth regulating factor protein | GO:0005654 nucleoplasm,  **GO:0000003 reproduction,**  GO:0006139 nucleobase, nucleoside, nucleotide and nucleic acid metabolic process,  GO:0003700 transcription factor activity,  **GO:0009791 post-embryonic development,**  **GO:0009790 embryonic development,**  GO:0009058 biosynthetic process  GO:0005634 nucleus,  GO:0007275 multicellular organismal development |
| Response to stimulus | osa-miR2118  osa-miR5497  osa-miR5509  osa-miR5528  osa-miR5534a  osa-miR5789  osa-miR5793  osa-miR5818 | NBS-LRR disease resistance protein  Ammonium transporter protein (LOC_Os01g61550.1)  Dirigent (LOC_Os10g18870.1)  S-adenosylmethionine synthetase (LOC_Os01g18860.1)  Katanin p80 WD40 repeat-containing subunit B1 homolog 1 (LOC_Os01g57210.1)  Anthocyanin 3-O-beta-glucosyltransferase (LOC_Os01g45140.1)  Dehydrin (LOC_Os11g26780.1)  Eukaryotic translation initiation factor (LOC_Os01g73880.1) | GO:0005737 cytoplasm,  **GO:0009607 response to biotic stimulus**,  GO:0000166 nucleotide binding,  GO:0006950 response to stress,  GO:0005886 plasma membrane,  GO:0008219 cell death  GO:0005215 transporter activity,  GO:0009987 cellular process,  GO:0006810 transport,  **GO:0009607 response to biotic stimulus**,  GO:0005886 plasma membrane  GO:0006950 response to stress,  GO:0005623 cell  GO:0006139 nucleobase, nucleoside, nucleotide and nucleic acid metabolic process,  **GO:0009628 response to abiotic stimulus**,  GO:0005618 cell wall,  GO:0005829 cytosol,  GO:0006950 response to stress,  GO:0009058 biosynthetic process,  GO:0016740 transferase activity,  GO:0005886 plasma membrane  GO:0009719 response to endogenous stimulus,  **GO:0009628 response to abiotic stimulus**,  GO:0000166 nucleotide binding,  GO:0006950 response to stress,  GO:0005886 plasma membrane,  GO:0005622 intracellular  GO:0005829 cytosol,  **GO:0009607 response to biotic stimulus**,  GO:0016740 transferase activity,  GO:0008152 metabolic process  GO:0009719 response to endogenous stimulus,  **GO:0009628 response to abiotic stimulus**,  GO:0006950 response to stress  GO:0005737 cytoplasm,  **GO:0009607 response to biotic stimulus**,  GO:0005515 protein binding,  GO:0005730 nucleolus,  GO:0008135 translation factor activity, nucleic acid binding,  GO:0003723 RNA binding,  GO:0034645 cellular macromolecule biosynthetic process,  GO:0006412 translation |
| Signalling | osa-miR5797 | Receptor-like protein kinase 2 precursor (LOC_Os11g36140.1) | GO:0044260 cellular macromolecule metabolic process,  GO:0016301 kinase activity,  GO:0006464 protein modification process,  GO:0000166 nucleotide binding,  GO:0023046 signalling process,  GO:0023052 signalling,  GO:0007165 signal transduction,  GO:0005886 plasma membrane,  GO:0023060 signal transmission |
| Development + Response to stimulus | osa-miR5487  osa-miR5492 | Mannose-6-phosphate isomerase (LOC_Os11g38810.1)  Auxin response factor  (LOC_Os04g57610.1)  Spotted leaf 11 (LOC_Os07g39590.1)  DUF647 domain containing protein (LOC_Os04g43690.1) | **GO:0009791 post-embryonic development,**  GO:0003824 catalytic activity,  GO:0005975 carbohydrate metabolic process,  GO:0000003 reproduction,  **GO:0009790 embryonic development**  **GO:0009719 response to endogenous stimulus,**  GO:0006139 nucleobase, nucleoside, nucleotide and nucleic acid metabolic process,  GO:0003700 transcription factor activity,  GO:0005634 nucleus,  GO:0009058 biosynthetic process,  **GO:0009908 flower development**  **GO:0009628 response to abiotic stimulus**,  GO:0009536 plastid,  GO:0005739 mitochondrion,  GO:0006810 transport |
| Response to stimulus + Signalling | osa-miR5488  osa-miR5491  osa-miR5791  osa-miR5792 | Hydroxymethylbutenyl 4-diphosphate synthase (LOC_Os02g39160.1)  Phytosulfokine receptor precursor (LOC_Os02g05950.1)  Resistance protein SlVe1 precursor (LOC_Os12g11500.1)  SAC9 (LOC_Os01g25330.1) | GO:0009536 plastid,  GO:0005975 carbohydrate metabolic process,  GO:0006629 lipid metabolic process,  GO:0009058 biosynthetic process,  GO:0023060 signal transmission,  GO:0003824 catalytic activity,  GO:0006950 response to stress,  GO:0023046 signalling process,  GO:0023052 signalling,  GO:0007165 signal transduction,  **GO:0009607 response to biotic stimulus**,  GO:0005488 binding  GO:0044260 cellular macromolecule metabolic process,  GO:0016301 kinase activity,  GO:0006464 protein modification process,  GO:0000166 nucleotide binding,  GO:0006950 response to stress,  GO:0023046 signalling process,  GO:0023052 signalling,  GO:0004872 receptor activity,  GO:0023060 signal transmission,  GO:0007165 signal transduction  GO:0009536 plastid,  GO:0016301 kinase activity,  GO:0023060 signal transmission,  GO:0006950 response to stress,  GO:0023046 signalling process,  GO:0023052 signalling,  GO:0007165 signal transduction,  GO:0008152 metabolic process  GO:0016787 hydrolase activity,  GO:0006629 lipid metabolic process,  **GO:0009628 response to abiotic stimulus,**  GO:0023060 signal transmission,  GO:0006950 response to stress,  GO:0023046 signalling process,  GO:0023052 signalling,  GO:0007165 signal transduction |
| Development + Response to stimulus + Signalling | osa-miR169f.2  osa-miR5517  osa-miR5485 | BRASSINOSTEROID INSENSITIVE 1-associated receptor kinase 1 precursor  (LOC_Os11g31560.1)  bZIP transcription factor  (LOC_Os01g64000.1)  Heat shock cognate 70 kDa protein 2  (LOC_Os12g38180.1)  KH domain containing protein (LOC_Os12g40560.1)  S-locus-like receptor protein kinase (LOC_Os03g30890.1)  Endothelial differentiation-related factor 1 (LOC_Os06g39240.1) | GO:0009719 response to endogenous stimulus,  GO:0044260 cellular macromolecule metabolic process, GO:0009838 abscission,  GO:0016301 kinase activity,  GO:0016043 cellular component organization,  GO:0023060 signal transmission,  **GO:0009790 embryonic development**,  GO:0023046 signalling process,  GO:0023052 signalling,  GO:0004872 receptor activity,  GO:0005886 plasma membrane,  GO:0006464 protein modification process,  GO:0005102 receptor binding,  **GO:0009908 flower development**,  GO:0007049 cell cycle,  GO:0007165 signal transduction  GO:0009719 response to endogenous stimulus,  **GO:0009628 response to abiotic stimulus**,  GO:0000003 reproduction,  GO:0006139 nucleobase, nucleoside, nucleotide and nucleic acid metabolic process,  GO:0003700 transcription factor activity,  GO:0005634 nucleus,  GO:0009058 biosynthetic process,  GO:0023060 signal transmission,  **GO:0009791 post-embryonic development**,  GO:0006950 response to stress,  GO:0023046 signalling process,  GO:0023052 signalling,  GO:0007165 signal transduction,  GO:0005515 protein binding  GO:0009628 response to abiotic stimulus,  GO:0009536 plastid,  **GO:0009607 response to biotic stimulus,**  GO:0005840 ribosome,  GO:0005730 nucleolus,  GO:0005773 vacuole,  GO:0005829 cytosol,  GO:0009987 cellular process,  GO:0000166 nucleotide binding,  **GO:0009791 post-embryonic development**,  GO:0019538 protein metabolic process,  GO:0006950 response to stress,  GO:0005576 extracellular region,  GO:0005515 protein binding,  GO:0005618 cell wall,  GO:0005886 plasma membrane,  GO:0043170 macromolecule metabolic process  GO:0005634 nucleus,  **GO:0009908 flower development,**  GO:0003723 RNA binding  GO:0009719 response to endogenous stimulus,  GO:0006139 nucleobase, nucleoside, nucleotide and nucleic acid metabolic process,  **GO:0009628 response to abiotic stimulus,**  GO:0003700 transcription factor activity,  GO:0005730 nucleolus,  GO:0009058 biosynthetic process,  GO:0023060 signal transmission,  GO:0006950 response to stress,  GO:0023046 signalling process,  GO:0023052 signalling,  GO:0007165 signal transduction |
