# Supplementary material for: Identification of functionally important microRNAs from rice inflorescence at heading stage of a qDTY4.1-QTL bearing Near Isogenic Line under drought conditions
Source: PLoS One. 2017 Oct 18;12(10):e0186382. doi: 10.1371/journal.pone.0186382 (PMC5647096; doi:10.1371/journal.pone.0186382)
Supplement: S1 Fig — IR = IR64, OFF = NIL (IR87705-7-15-B) and P1 = BIL (IR77298-14-1-2-10) rice lines; C = control and D = drought-treated conditions. (DOCX) [file pone.0186382.s005.docx]

**S1 Fig**

**RNA quality: RNA integrity (RIN) analysis.**


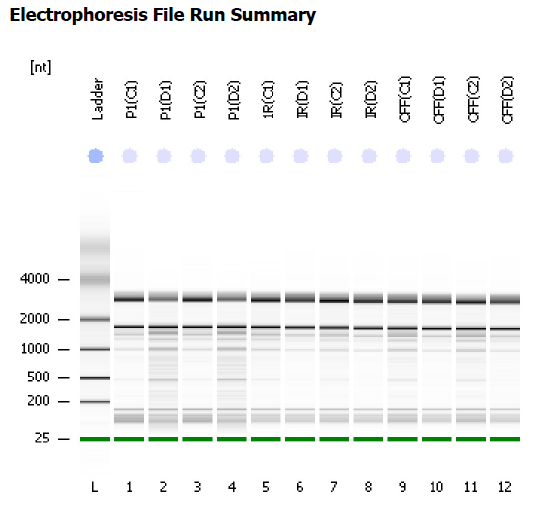


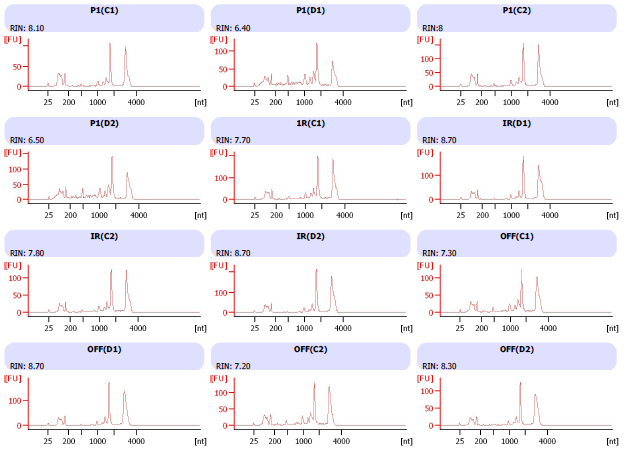
IR = IR64, OFF = NIL (IR87705-7-15-B) and P1 = BIL (IR77298-14-1-2-10) rice lines

C = control and D = drought-treated conditions.
