# Supplementary material for: Identification of functionally important microRNAs from rice inflorescence at heading stage of a qDTY4.1-QTL bearing Near Isogenic Line under drought conditions
Source: PLoS One. 2017 Oct 18;12(10):e0186382. doi: 10.1371/journal.pone.0186382 (PMC5647096; doi:10.1371/journal.pone.0186382)
Supplement: S2 Fig — NIL = IR87705-7-15-B and BIL = IR77298-14-1-2-10 rice lines. (DOCX) [file pone.0186382.s006.docx]

**S2 Fig**

**PCA plot and heatmap was generated with normalized expression of miRNAs from the six samples.**

**(A) PCA plot**


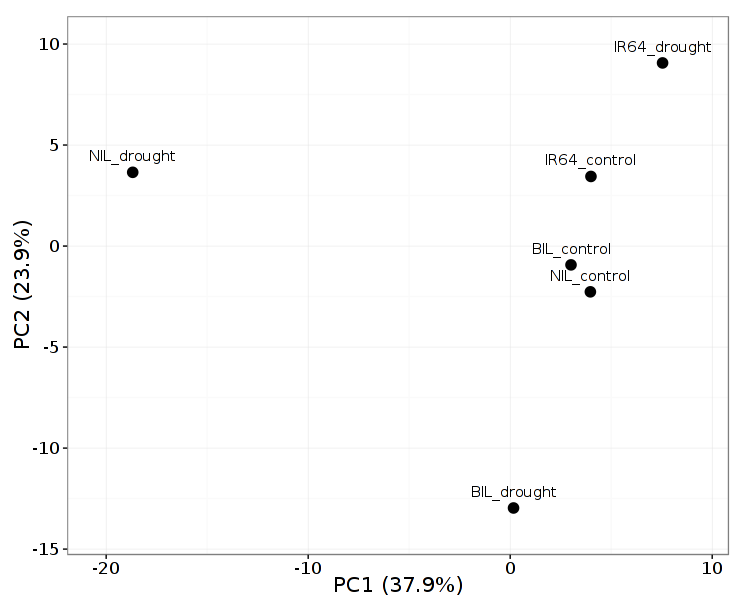


**(B) Heatmap**


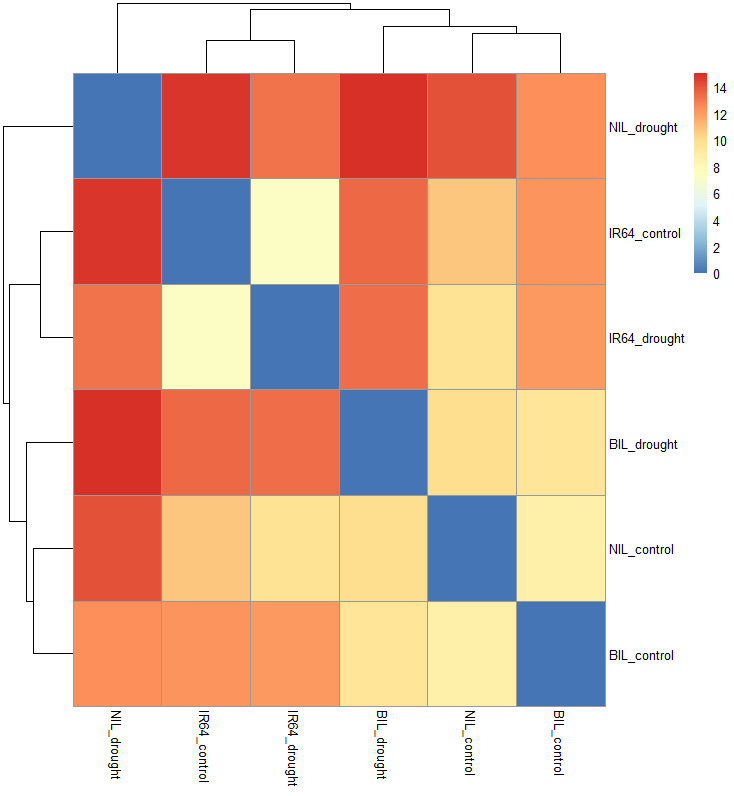
NIL = IR87705-7-15-B and BIL = IR77298-14-1-2-10 rice lines.
